# Computational Method Using Attribute-Aware Message Passing and Graph Convolutional Network for Potential miRNA–Disease Association Prediction

**DOI:** 10.3390/ijms27136077

**Published:** 2026-07-07

**Authors:** Peng Qin, Jiyong An

**Affiliations:** 1 School of Computer Science, Liupanshui Normal University, Liupanshui 553004, China; 2 School of Computer Science and Technology, China University of Mining and Technology, Xuzhou 221116, China

**Keywords:** MDAs, attribute-aware message passing, GCN, multi-subgraph embedding fusion

## Abstract

MicroRNA (miRNA) dysregulation is a crucial pathogenic factor that extensively participates in the occurrence and progression of various human diseases, especially cancers. Identifying unknown miRNA–disease connections is essential for understanding disease pathogenesis and improving clinical treatment strategies. Traditional biological experiments are often expensive and technically restricted, so computational prediction has become a widely used auxiliary research tool. In this study, we develop a novel predictive model called Attribute-Aware Message Passing Graph Convolutional Network (AAMPGCN) to identify potential miRNA–disease associations. The advantage of AAMPGCN lies in integrating miRNA and disease attribute information into the message-passing process: it partitions the miRNA–disease heterogeneous graph that incorporates miRNA functional similarity, disease semantic similarity, and Gaussian interaction kernel similarity into attribute-homogeneous subgraphs, while restricting high-order message propagation within each subgraph. This mechanism effectively filters cross-attribute noise, preserves the discriminability of miRNA and disease embeddings during deep convolution, and is thus well-adapted to miRNA–disease heterogeneous networks. The AAMPGCN prioritizes miRNA and disease attributes, aggregating messages specifically among nodes with similar attribute characteristics that are relevant to miRNA–disease interactions. Experimental results show that the AAMPGCN model achieves AUC and AUPR values of 94.06 and 93.52 on the HMDD2.0 dataset, which outperforms existing methods. The proposed AAMPGCN provides a new and effective method for miRNA–disease association prediction, and also offers theoretical support for the research on disease molecular mechanisms and the screening of clinical therapeutic targets.

## 1. Introductions

MicroRNAs (miRNAs) are a class of endogenous non-coding RNA molecules, typically 18–25 nucleotides in length, that play pivotal regulatory roles in a broad spectrum of cellular and molecular processes [1,2,3,4]. These processes include, but are not limited to, cell cycle progression, cellular differentiation, programmed cell death (apoptosis), and intercellular signal transduction [5,6,7]. Over the past decade, accumulating experimental evidence has demonstrated that perturbations in miRNA expression profiles or functional dysregulation are closely correlated with the initiation, progression, and clinical prognosis of various human pathological conditions, including malignant tumors, cardiovascular disorders, and neurodegenerative diseases. By modulating the post-transcriptional expression of target genes, miRNAs are actively involved in the regulation of key biological pathways; their aberrant associations with diseases often disrupt the homeostasis of gene regulatory networks, ultimately contributing to the development and progression of pathological phenotypes [8,9,10,11].

Identifying the intricate associations between miRNAs and diseases is fundamental to deciphering the molecular mechanisms underlying disease pathogenesis, developing sensitive early diagnostic biomarkers, and identifying potential therapeutic targets for precision medicine. Nevertheless, conventional experimental techniques employed to validate miRNA–disease associations—such as luciferase reporter assays, PCR and RNA sequencing are inherently associated with high costs, prolonged experimental cycles, and intensive labor requirements [12,13,14,15]. These inherent limitations pose a significant challenge in keeping pace with the exponential growth of miRNA and disease-related omics data, leading to a substantial number of unconfirmed potential miRNA–disease associations that remain unexplored and require further investigation [16,17]. To address this bottleneck, computational prediction methods have been extensively developed and applied as an effective complement to traditional experimental verification, greatly accelerating the discovery of potential miRNA–disease associations.

At present, the existing computational prediction methods for miRNA–disease associations can be roughly divided into several categories, including similarity-based methods, matrix completion-based methods, machine learning-based methods, and graph neural network-based methods, each with its own unique advantages and inherent deficiencies [18,19,20]. Similarity-based methods are simple and interpretable, enabling rapid preliminary screening without complex training, but they often rely on single-type similarity information, suffer from inaccurate predictions when similarity data is sparse, and fail to capture complex non-linear relationships. For instance, Cui et al. [21] proposed a method to construct pairwise functional similarities and functional networks of human miRNAs via disease association structures. Comparative results demonstrate that the obtained miRNA functional similarities are consistent with known miRNA functional links. Chen et al. [12] proposed three miRNA–disease association prediction approaches: MBSI (microRNA-based similarity inference), PBSI (phenotype-based similarity inference), and NetCBI (network-consistency-based inference). All three leverage global network similarity metrics to identify unreported miRNA–disease pairs. Chen et al. [22] developed a WBSMDA (Within and Between Score for MiRNA–Disease Association prediction) to identify potential miRNA-complex disease associations. This model works for diseases lacking known relevant miRNAs, and leave-one-out cross-validation yields an AUC of 0.8031, verifying its stable predictive capacity. Le et al. [23] adopt a customized random walk with restart algorithm to infer disease-miRNA associations, utilizing complementary information from validated/predicted miRNA-target interactions and disease phenotypic similarities. Matrix completion-based methods excel at handling sparse association matrices and mining hidden patterns, yet they have high computational complexity, struggle with cold-start problems, and ignore node attribute differences. For instance, Chen et al. [24] propose IMCMDA (Inductive Matrix Completion for MiRNA–Disease Association prediction). We compute integrated miRNA and disease similarities from miRNA functional similarity, disease semantic similarity, and Gaussian interaction profile kernel similarity. The model recovers unobserved miRNA–disease associations using known links and the above integrated similarities. Chen et al. [25] propose LRMCMDA, a low-rank matrix completion method for miRNA-disease association prediction. It first builds a bipartite miRNA–disease graph from known associations and constructs an R-projected miRNA graph where edges connect miRNAs sharing common disease neighbors. A corresponding D-projected disease graph is defined analogously. Li et al. [26] construct AMCMDA (Accurate Matrix Completion Model) for latent miRNA–disease association prediction, which improves prediction accuracy via truncated nuclear norm minimization. AMCMDA first builds a heterogeneous network integrating miRNA similarities, disease similarities and known associations. Next, an optimization framework is devised to approximate the truncated nuclear norm and recover missing entries of the association matrix. Finally, the alternating direction method of multipliers (ADMMs) is adopted to solve the optimization and output prediction scores, Qin et al. [27] propose EMCMDA (Efficient Matrix Completion for MiRNA–Disease Association prediction) to infer miRNA–disease associations. First, we compute multi-source similarities for miRNAs and diseases and fuse them into unified similarity metrics. Second, we construct a heterogeneous network with these biological similarities and build the target association matrix. Finally, we formulate association prediction as a low-rank matrix completion problem solved by minimizing the truncated Schatten p-norm. Traditional machine learning methods exhibit good adaptability to different data types and possess certain generalization ability. Traditional machine learning-based methods adapt well to different data types and have certain generalization ability. For instance, Wei L. et al. [28] proposed a novel DFELMDA model that first constructs multi-type feature representations for miRNAs and diseases, and adopts two deep autoencoders to extract low-dimensional latent features. Deep random forest is then used to generate and fuse prediction scores for final inference. Chen X. et al. [29] propose EGBMMDA, an extreme gradient boosting machine model for miRNA–disease association prediction and the first decision tree learning-based model for such a prediction task. Qiguo D. et al. [30] present ERMDA, a resampling-based ensemble framework for miRNA–disease association prediction. It constructs balanced training subsets to alleviate sample imbalance and extracts miRNA and disease features using multi-source similarity and verified association information. Kai Z. et al. [31] present a machine learning model called MLMDA that uses a random forest classifier for miRNA–disease association prediction. Graph neural network-based methods effectively capture complex network association patterns and integrate topological and attribute information. For instance, Wang S. et al. [32] propose a heterogeneous graph attention network based on meta-subgraphs called MSHGANMDA that first define five meta-subgraph types from known associations, then use meta-subgraph attention and semantic attention to extract features of miRNA–disease pairs within and between these meta-subgraphs, and, finally, apply a fully connected layer to predict association scores and cross-entropy loss for end-to-end training. Han H. et al. [33] adopts graph convolutional networks to learn feature embeddings of miRNAs and diseases, and introduces an attention mechanism to fuse embeddings output from multiple convolutional layers. Finally, based on the fused embeddings, scores are predicted for unknown miRNA–disease associations. Gao S. et al. [34] proposed a GCN-based model DEJKMDR for miRNA–disease association prediction, integrating biomolecular information and Gaussian interaction profile kernel similarity to avoid overfitting, with adaptive weight allocation for feature fusion and accurate association scoring. Liang X. et al. [35] proposed a novel method for miRNA–disease association prediction, which integrates a graph convolutional network and a hypergraph convolutional network. The graph convolutional network extracts feature from miRNA and disease similarity data, while the hypergraph convolutional network captures complex high-order interactions of known miRNA–disease associations based on the learned representations. In summary, although existing computational methods for miRNA–disease association prediction have their respective advantages and have achieved remarkable progress in assisting biological research, there is still room for improvement in feature utilization, computational efficiency, and sparse data processing. Therefore, it is urgent to develop a novel computational model that can fully integrate multi-source information and improve prediction accuracy and robustness.

In this study, we develop a novel predictive model called Attribute-Aware Message Passing Graph Convolutional Network (AAMPGCN) to identify potential miRNA–disease associations. The advantage of AAMPGCN lies in integrating miRNA and disease attribute information into the message-passing process: it partitions the miRNA–disease heterogeneous graph that incorporates miRNA functional similarity, disease semantic similarity, and Gaussian interaction kernel similarity into attribute-homogeneous subgraphs, while restricting high-order message propagation within each subgraph. This mechanism effectively filters cross-attribute noise, preserves the discriminability of miRNA and disease embeddings during deep convolution, and is thus well-adapted to miRNA–disease heterogeneous networks. The AAMPGCN prioritizes miRNA and disease attributes, aggregating messages specifically among nodes with similar attribute characteristics that are relevant to miRNA–disease interactions. Experimental results show that the AAMPGCN model achieves AUC and AUPR values of 94.06 and 93.52 on the HMDD2.0 dataset, which outperforms existing methods. The proposed AAMPGCN provides a new and effective method for miRNA–disease association prediction, and also offers theoretical support for the research on disease molecular mechanisms and the screening of clinical therapeutic targets.

## 2. Results

### 2.1. Performance of the Proposed Method

To fully assess the effectiveness of the proposed AAMPGCN model in predicting miRNA–disease associations (MDAs), we carried out strict experiments on the HMDD v2.0 dataset, which is widely used in MDA prediction research and contains 495 miRNAs, 383 diseases, and 5430 known miRNA–disease associations. To maintain class balance, an equal number of negative samples was randomly extracted from unlabeled association pairs. The 5-fold cross-validation (5-CV) method was employed to ensure the credibility of the experimental results and avoid overfitting.

**Figure 1:**
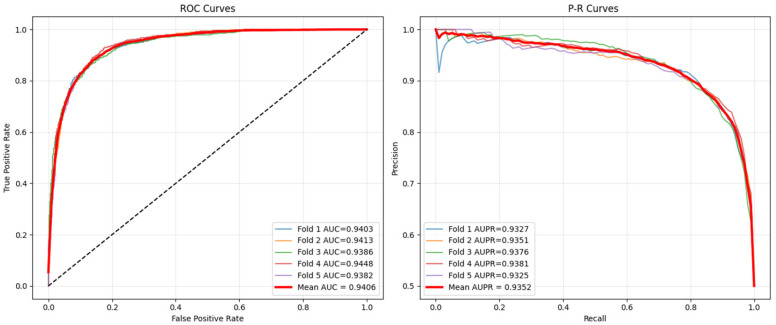
ROC and P-R plots for AAMPGCN trained on HMDD v2.0.

The proposed AAMPGCN model was built based on PyTorch 2.0 and trained for over 1000 epochs. After parameter tuning, the learning rate was set to 0.0006 with a dropout rate of 0.4. Several classic classification metrics were adopted to assess model performance, and the corresponding quantitative results and ROC, as well as PR curves, are presented in Table 1 and Figure 1, respectively. Figure 1 shows the Receiver Operating Characteristic (ROC) curves and Precision–Recall (PR) curves plotted in this work, which quantitatively characterize the predictive performance of our proposed model. The ROC curve plots true positive rate (TPR) against false positive rate (FPR) under varied classification thresholds. The area enclosed by the ROC curve (AUC) reflects the model’s general discrimination capacity: an AUC close to 1 implies nearly perfect classification, whereas an AUC of 0.5 equals random guesses. Considering the severe class imbalance inherent in miRNA–disease datasets, ROC evaluation alone may overestimate model performance. Therefore, we further adopted PR curves as a complementary assessment metric. The PR curve depicts Precision versus Recall, and the corresponding AUPR value provides a more reliable evaluation for imbalanced biological data. Figure 1 illustrates that our proposed approach obtains an average AUC value of 94.06 on the ROC curve and an average AUPR value of 93.52 on the PR curve, exceeding all baseline models, which further demonstrates the reliability and superiority of the model developed in this work.

### 2.2. Comparison with Other Models

To further explore the prediction performance of AAMPGCN in identifying miRNA–disease associations (MDAs), we compared our method with several state-of-the-art computational models:

1. HHOMR [36]: This model exploits high-order statistical characteristics and graph structural information, and adopts an attention mechanism to effectively mine implicit correlation patterns between miRNAs and diseases.
2. MTLMDA [37]: It integrates miRNA–disease and gene–disease networks through cross-layer compression, employs graph convolutional networks to extract node features, and utilizes a linear decoder to complete association prediction.
3. VGAEMDA [38]: The model leverages the variational graph autoencoder framework to conduct unsupervised representation learning, thereby capturing underlying miRNA–disease association relationships.
4. GBDT-LR [39]: It first adopts matrix factorization to acquire low-dimensional embedding representations of the miRNA–disease adjacency matrix, and then excavates potential association dependencies under a label space learning paradigm.

**Figure 2:**
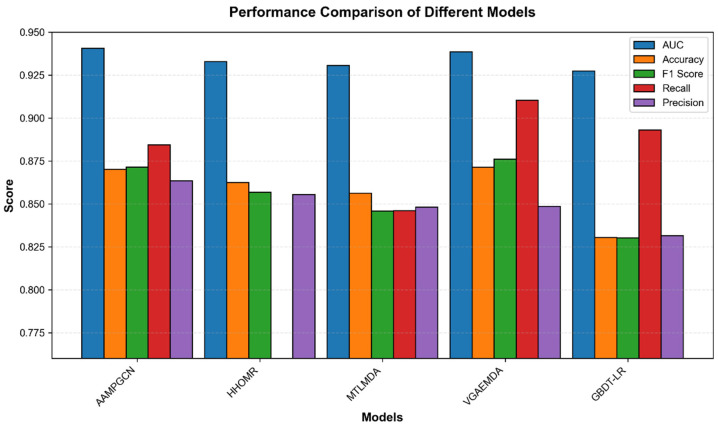
Bar Chart for Comparative Evaluation of Model Performance.

Comparisons among the aforementioned models and our developed model are summarized in Table 2 and Figure 2.

As illustrated in Figure 2 and Table 2, our model outperforms all competing methods, mainly for the following reasons: the AAMPGCN integrates three biologically meaningful similarity matrices into a unified graph learning framework. The AAMPGCN integrates miRNA and disease attribute information into the message-passing process, partitioning attribute-homogeneous subgraphs, and restricting high-order message propagation within subgraphs, which effectively filters cross-attribute noise, avoids over-smoothing, and maintains the discriminability of node embeddings. Moreover, the AAMPGCN prioritizes miRNA and disease attributes, aggregating messages specifically among nodes with similar attribute characteristics that are relevant to miRNA–disease interactions. This is the core reason why AMPGCN outperforms other state-of-the-art methods in MDA prediction.

### 2.3. Hyperparameter Sensitivity Analysis

To improve the prediction performance of the proposed AAMPGCN model and ensure its stability and generalization ability, hyperparameter optimization experiments were carried out, focusing on two key hyperparameters that significantly influence the model’s feature learning and representation capabilities: the polynomial order K of graph convolutional layers and the hidden layer dimension. All experiments were implemented based on the PyTorch framework, with consistent training settings (1000 epochs, learning rate of 0.0006, dropout rate of 0.4, and Adam optimizer with weight decay of 1 × 10^−4^) to isolate the impact of the two target hyperparameters on model performance. The model’s performance was evaluated using the AUC and AUPR values as the primary metric to ensure the comprehensiveness and reliability of the evaluation results. The polynomial order K is a crucial hyperparameter that determines the scope of local neighborhood aggregation during the feature learning process. A smaller K value may lead to insufficient extraction of neighborhood information, resulting in underfitting and limited model capacity; on the contrary, an excessively large K value may introduce redundant noise from distant nodes, increase model variance, and further cause overfitting. To determine the optimal value of K, a grid search was performed within the range of 1 to 5. Experimental results demonstrated that the model achieved the best comprehensive performance when K = 3, with an average AUC of 0.9415. When K = 1 and K = 2, the model only aggregated information from directly adjacent nodes, leading to inadequate feature learning and a relatively low AUC and AUPR. For K ≥ 3, the model aggregated information from more distant nodes, which resulted in increased noise interference and a slight decrease in AUC and AUPR for K = 4 and K = 5. This confirms that K = 3 can achieve an optimal balance between model capacity and stability.

The hidden layer feature dimension is another core hyperparameter that affects the model’s ability to learn high-dimensional feature representations of miRNA and disease nodes. A too-small feature dimension may restrict the model’s feature learning capacity, making it difficult to capture the complex latent relationships between miRNAs and diseases; a too-large feature dimension may increase the model’s computational complexity, induce overfitting, and reduce the model’s generalization ability. A grid search was conducted for the feature dimension within the range of 32, 64, 128, and 256, which are widely used in graph neural network models for miRNA–disease association prediction. Experimental results showed that the model achieved the highest AUC of 0.9421 when the feature dimension was set to 128. When the feature dimension was 32 and 64, the model’s feature representation ability was insufficient, resulting in a low AUC. When the feature dimension was increased to 256, although the model’s feature learning capacity was enhanced, it led to overfitting, and the AUC decreased to 0.8636. Therefore, 128 was determined as the optimal feature dimension for AAMPGCN, which can effectively balance the feature representation ability and computational efficiency.

**Figure 3:**
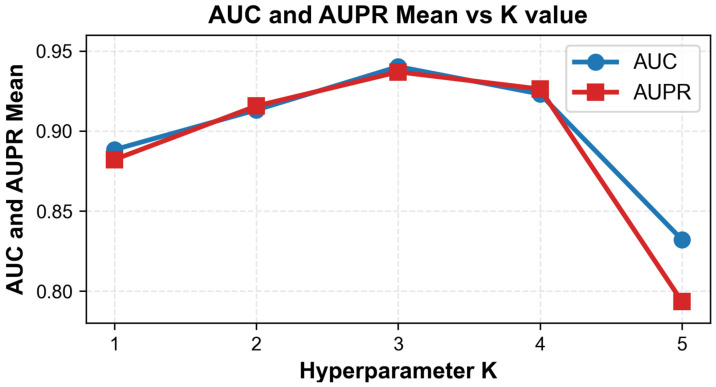
Result of employing different k values in AAMPGCN.

**Figure 4:**
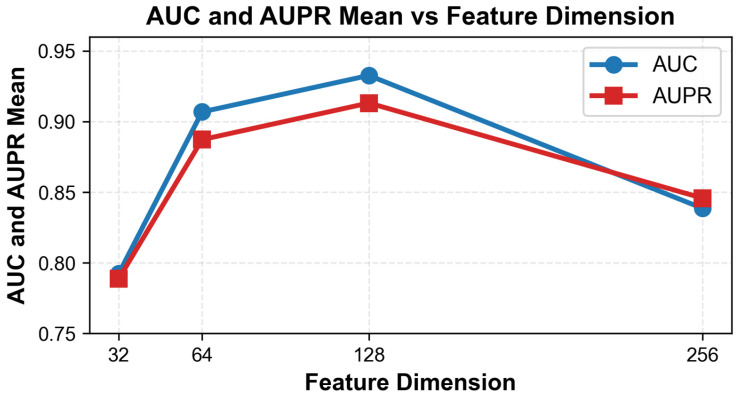
Result of employing different feature dimensions in AAMPGCN.

In summary, the optimal hyperparameters of AAMPGCN determined through the above experiments are as follows: the polynomial order K = 3 of graph convolutional layers and the hidden layer dimension = 128. These optimal hyperparameters enable the model to effectively aggregate local neighborhood information, learn discriminative feature representations, and thus achieve excellent performance in miRNA–disease association prediction. The comparison of AUC and AUPR under different values of the two hyperparameters is presented in Figure 3 and Figure 4.

### 2.4. Case Study

The AAMPGCN method was used to test the accuracy of predictive associations via three cases (prostate, lung, and pancreatic cancer), with predicted candidate miRNAs verified against the DBDEMC [40] and Phenomir databases [41].

**Figure 5:**
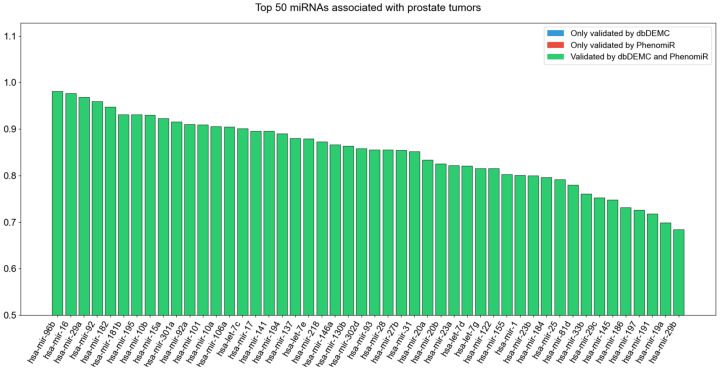
Prediction of top 50 miRNAs related to prostate tumors.

**Figure 6:**
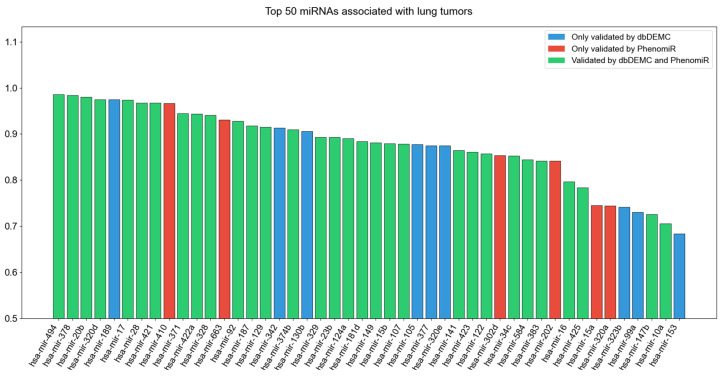
Prediction of top 50 miRNAs related to lung tumors.

**Figure 7:**
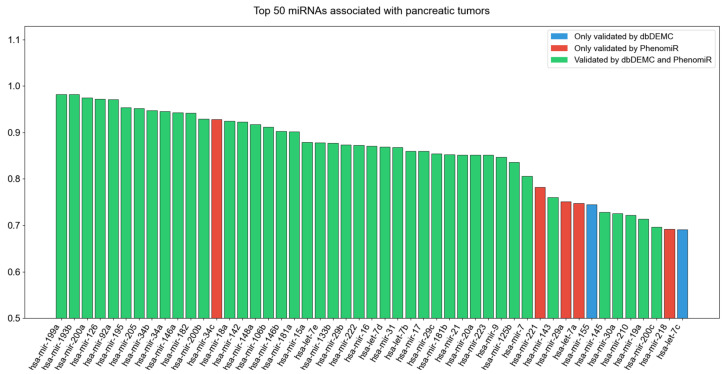
Prediction of top 50 miRNAs related to pancreatic tumors.

In the first case, prostate neoplasms, which are the most common male malignancy globally and cause over 100,000 European male deaths in 2018 [42], were selected to test AAMPGCN’s applicability to novel diseases without known associated miRNAs. All miRNA–disease associations for prostate neoplasms in HMDD 2.0 were set to zero, followed by the application of AAMPGCN to identify associated miRNAs. Figure 5 lists AAMPGCN’s top 48 candidate miRNAs for prostate neoplasms, all of which were verified by the DBDEMC and Phenomir databases. Notably, the first-ranked hsa-miR-96b regulates prostate cancer cell apoptosis by inhibiting FoxO1, thereby validating AAMPGCN’s predictive ability for such novel diseases. For the second case, we conducted a study on lung neoplasms, which is a fatal global cancer with a low five-year survival rate [43] and a disease where miRNAs assist in diagnosis and treatment. We adopted the same data processing method as that used in the first case. Among AAMPGCN’s top 46 predicted miRNAs for lung neoplasms listed in Figure 6, 46 were validated by one or both of the two databases. Ectopic expression of miR-494 in A549 lung cancer cells promoted tumor-initiating cells, enhanced cell motility, and upregulated the expression of stem cell-related genes, supporting AAMPGCN’s value for the diagnosis and treatment of lung cancer. For the third case, pancreatic tumors were selected as the new disease, and the same experimental method as that used in the previous two cases was adopted. The top 49 predicted results are shown in Figure 7, all of which have been verified by one or both of the two databases. Existing studies have shown that increased serum miR-193b expression can serve as a potential new biomarker for pancreatic neuroendocrine tumors (PNEN).

The experimental results of the above three cases further demonstrate the important role of the AAMPGCN model in the prediction of new diseases.

To further verify the effectiveness of AAMPGCN, we compared the counts of database-validated miRNA–disease associations among the top 50 prediction candidates generated by our model and other competing baselines. Specifically, we performed this statistical comparison on two representative diseases covered in this work, namely lung cancer and prostate cancer, by tallying the total number of biologically confirmed miRNA–disease pairs ranked within the top 50 predictions for each competing method. All comparative outcomes are summarized in Table 3.

As shown in Table 3, AAMPGCN identifies more validated miRNA–disease associations than all competing prediction models, further verifying its superior predictive performance. This advantage mainly stems from the unique architecture of AAMPGCN. The model incorporates attribute features of miRNAs and diseases into the message-passing scheme to build a high-quality heterogeneous graph fused with multiple similarity measurements. Meanwhile, it constrains high-order message propagation within attribute-homogeneous subgraphs. This tailored design effectively eliminates cross-attribute noise and mitigates the ubiquitous over-smoothing problem of traditional graph convolutional networks. Consequently, high-discriminative node embeddings are learned, enabling the model to precisely capture inherent biological correlations between miRNAs and diseases.

## 3. Discussion

Our proposed Attribute-Aware Message Passing Graph Convolutional Network (AAMPGCN) demonstrates superior performance in MDA prediction, which is fully validated by systematic experiments and case studies. Experiments were conducted on the HMDD 2.0 dataset and used a 5-fold cross-validation strategy to ensure the reliability and generalizability of the results and to avoid overfitting. Key evaluation metrics included Area Under the Receiver Operating Characteristic Curve (AUC), Area Under the Precision–Recall Curve (AUPR), Accuracy (ACC), Precision, Recall, and F1-score, among which AUC and AUPR were the primary indicators to reflect the model’s predictive ability. AAMPGCN achieved excellent performance across all metrics: an AUC of 0.9406, an AUPR of 0.9352, an ACC of 0.8702, an F1-score of 0.8715, and a precision of 0.8635. This performance significantly outperformed mainstream baseline models, including HHOMR, MTLMDA, VGAEMDA, and GBDT-LR.

The excellent experimental performance of AAMPGCN can be attributed to its core design features, which are specifically tailored to the characteristics of miRNA–disease heterogeneous networks. Specifically, AAMPGCN integrates miRNA and disease attribute information into the message-passing process, constructs a heterogeneous graph incorporating miRNA functional similarity, disease semantic similarity, and Gaussian interaction kernel similarity, and restricts high-order message propagation to attribute-homogeneous subgraphs. This design effectively filters out cross-attribute noise, avoids the over-smoothing problem that plagues traditional graph convolutional networks, and ensures the generation of high-quality node embeddings, thereby enabling the model to accurately capture the intrinsic interactions between miRNAs and diseases.

Case studies on prostate, lung, and pancreatic cancer—three common malignant tumors closely associated with miRNA dysregulation—further validated the practical value of AAMPGCN. Among the top 50 predicted MDAs for each disease, all were supported by authoritative databases or the published experimental literature.

In summary, AAMPGCN’s superior experimental performance confirms its reliability as a powerful computational tool for MDA research, providing valuable support for subsequent biomedical experiments and clinical research.

Despite AAMPGCN’s promising performance in miRNA–disease association (MDA) prediction, it has several limitations to address in future work:

- Dependence on similarity matrix quality: Its performance is closely linked to the quality and coverage of input similarity matrices, but incomplete annotations in existing biological databases (e.g., insufficient information for rare miRNAs/diseases) lead to inaccurate similarity calculations and compromised performance.
- Lack of regulatory pathway modeling: It focuses on pairwise miRNA–disease associations but fails to explicitly model intermediate regulatory mechanisms (e.g., miRNA–gene–disease networks), which limits model interpretability.
- Transudative learning limitation: As a transudative model, it can only predict for miRNAs/diseases in the training set and cannot generalize to unseen nodes without retraining, restricting practical application in emerging disease research.

To address these issues, future work will focus on three directions: (1) Integrate multi-omics data to construct more comprehensive and accurate miRNA/disease similarity metrics, reducing the impact of incomplete annotations. (2) Design an inductive graph learning module (via meta-learning or contrastive learning) to enable generalization to unseen miRNAs/diseases without retraining. (3) Incorporate pathway-level attention mechanisms to model miRNA–gene–disease regulatory pathways, enhancing model interpretability and providing deeper mechanistic insights.

## 4. Materials and Methods

### 4.1. Datasets

The Human microRNA Disease Database (HMDD2.0) is a well-known benchmark database that collects experimentally verified miRNA–disease associations. The HMDD2.0 includes 495 miRNAs, 383 diseases, and 5430 confirmed associations [45]. To facilitate subsequent analysis, we constructed an adjacency matrix $A\in R^{n_{d}\times n_{m}}$ to formalize the associations between miRNAs and diseases. Here, $n_{m}$ and $n_{d}$ denote the number of known miRNAs and known diseases, respectively. Specifically, if the disease $d_{i}$ has been experimentally verified to be associated with the miRNA $m_{j}$, the element $A_{ij}$ is set to 1; otherwise, it is 0.

### 4.2. miRNA Functional Similarity

Based on the hypothesis that miRNAs with analogous functions tend to be associated with phenotypically similar diseases [21]. In this study, the functional similarity information for miRNAs FS, obtained from the MISIM (miRNA similarity) resource, we used matrix *FS* (miRNA functional similarity) to characterize miRNA functional similarity, where the entry $\mathrm{FS}(m_{i},m_{j})$ denotes the similarity score between miRNA $m_{i}$ and miRNA $m_{j}$. The final *FS* value is calculated via cosine similarity, as shown in Equation (1):

(1) $$FS\left(m_{1},m_{2}\right)={COS}_{mir}\left(m_{1},m_{2}\right)=\frac{f_{m1}\cdot f_{m2}^{T}}{{||f}_{m1}|\left|\cdot {||f}_{m2}\right||}$$

where $f_{m1}$ and $f_{m2}$ represent enhanced disease semantic feature vectors of *miRNA* $m_{1}$ and miRna $m_{2}$, respectively; $f_{m1}\cdot f_{m2}^{T}$ represents the dot product of two vectors and ||f|| represents the L2 norm of the vector.

### 4.3. Disease Semantic Similarity

Disease semantic similarity is established relying on the Medical Subject Headings (MeSH) database [46,47,48]. The internal connections among various diseases can be intuitively abstracted through a Directed Acyclic Graph (*DAG*). Specifically, the topological structure of disease *D* is defined as *DAG*(*D*) = (*T*(*D*), *E*(*D*)). In this definition, *T*(*D*) refers to the node collection covering the target disease D and all its ancestral nodes, while *E*(*D*) stands for the edge set connecting these associated nodes. For an arbitrary disease node d contained in *DAG*(*D*), the semantic contribution is as follows [49]:

(2) $$D1_{D}\left(d\right)=\left\{\begin{array}{c} 1\;\;\;\;\;\;\;\;\;\;\;\;\;\;\;\;\;\;\;\;\;\;\;\;\;\;\;\;\;\;\;\;\;\;\;\;\;\;\;\;\;\;\;\;\;\;\;\;\;\;\;\;\;\;\;\;\;\;\;\;\;\;\;\;\;\;\;\;\;\;\;if\;d\;=\;D\;\\ \max\{\Delta \cdot D1_{D}(d^{\prime })\;|\;d^{\prime }\;\in \;\mathrm{children}\;\mathrm{of}\;d\},\;\;if\;d\;\neq \;D, \end{array}\right\}$$

where Δ represents the semantic contribution decay factor, usually set to 0.5 [47]. The semantic similarity of disease *D* is defined as

(3) $$DS1\left(D\right)=\sum_{d\in T(D)}D1_{D}\left(d\right)$$

The semantic contribution score described above is represented by *SS*1, where each element *SS*1(*i*,*j*) is computed via the following formula:

(4) $$SS1\left(i,j\right)=\frac{\sum_{t\in T(i)⋂T(j)}\left({D1}_{i}\left(t\right)+{D1}_{j}\left(t\right)\right)}{DS1\left(i\right)+DS1\left(j\right)}$$

From Equations (1) and (2), it can be observed that within the DAG structure of disease D, the semantic contribution of the root node *D* itself is defined as 1. As the topological distance from *D* to its ancestor nodes increases, the semantic contribution of these nodes to D decays progressively. It is also clear that nodes located at the same hierarchical level share identical semantic contribution scores. Considering that nodes unique to *DAG*(*D*) tend to contribute more to the semantics of disease *D* than those shared across multiple disease DAGs, Xuan et al. [37] developed an alternative computational model, as described below.

(5) $$DS2\left(D\right)=-\log\left(\frac{the\;numbers\;of\;DAGs\;includ\;d}{the\;numbers\;of\;diseases}\right)$$

Correspondingly, the alternative semantic contribution measure is formulated as follows:

(6) $$DS2\left(D\right)=\sum_{d\in T(D)}D2_{D}\left(d\right)$$

(7) $$SS2\left(i,j\right)=\frac{\sum_{t\in T(i)⋂T(j)}\left({D2}_{i}\left(t\right)+{D2}_{j}\left(t\right)\right)}{DS2\left(i\right)+DS2\left(j\right)}$$

In conclusion, the disease semantic similarity is defined by the formula presented below:

(8) $$SS\left(i,j\right)=\frac{ss1\left(i,j\right)+ss2(i,j)}{2}$$

### 4.4. Gaussian Interaction Profile Kernel Similarity

As a widely adopted measurement for assessing entity similarity, the Gaussian interaction profile (GIP) kernel similarity is extensively applied in relevant research. The GIP kernel for miRNAs can be computed via Equations (2)–(8) [50,51,52]:

$$GM\left(i,j\right)=exp(-\gamma_{m}||A\left(m_{i}\right)-A\left(m_{j}\right){\left|\right|}^{2})$$

in which $A\left(m_{i}\right)$ refers to the association profile of miRNA *i*, while $A\left(m_{j}\right)$ corresponds to that of miRNA *j*. $\gamma_{m}$ serves as a tunable parameter for kernel bandwidth optimization, with the specific calculation equation shown below:

(9) γm=γm′(1nm∑i=1nm||A(mi)||2)

in which $\gamma_{m}^{\prime }$ is assigned a fixed value of 1. Likewise, the Gaussian interaction profile kernel for diseases is formulated as

(10) $$GD\left(i,j\right)=exp(-\gamma_{d}||A\left(d_{i}\right)-A\left(d_{j}\right){\left|\right|}^{2})$$

(11) γd=γd′(1nd∑i=1nd||A(di)||2)

in which $A\left(d_{i}\right)$ and $A\left(d_{j}\right)$ denote the interaction profiles corresponding to disease *I* and disease *j*, with $\gamma_{d}^{\prime }$ assigned as 1.

### 4.5. Joint Similarity Kernel of miRNAs and Diseases

In this work, we construct a miRNA–disease joint similarity kernel to fuse multiple biological similarity information. Concretely, by combining the functional similarity kernel (*FS*) and the Gaussian interaction profile kernel (*GM*) of miRNAs, the comprehensive miRNA similarity kernel *SM* can be established. The definition of this fused similarity kernel is given below:

(12) $$SM\;=\left\{\begin{array}{c} FS\left(i,j\right),\;\;\;\;\;\;\;\;\;\;\;FS(i,j)\;\neq 0\\ GM\left(i,j\right),\;\;\;\;\;\;\;\;\;\;\;\;\;\;\mathrm{otherwise} \end{array}\right\}$$

According to the above definition, the Gaussian interaction profile kernel *GM* is utilized to fill the missing values of the functional similarity matrix *FS* in the fused similarity kernel. In a similar manner, the disease similarity kernel *SD* is formulated as

(13) $$SD=\left\{\begin{array}{c} SS\left(i,j\right),\;\;\;\;\;\;\;\;\;\;\;SS(i,j)\;\neq 0\\ GD\left(i,j\right),\;\;\;\;\;\;\;\;\;\;\;\;\;\;\mathrm{otherwise} \end{array}\right\}$$

### 4.6. Attribute-Aware Message Passing GCN(AAMPGCN)

**Figure 8:**
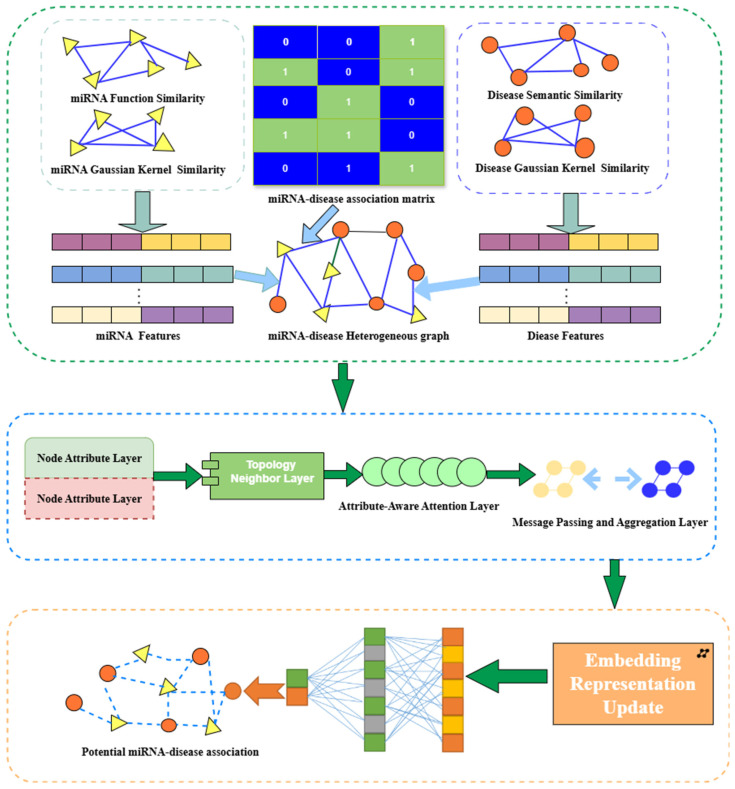
Flowchart of AAMPGCN model for predicting MDAs. Figure 8 illustrates the complete technical pipeline of our proposed AAMPGCN for miRNA-disease association prediction. The detailed descriptions are as follows: The green box corresponds to Step 1: Heterogeneous Graph Construction. We construct a bipartite miRNA-disease graph and derive multi-view node attribute features from miRNA functional similarity, disease semantic similarity and Gaussian kernel similarity. The blue box corresponds to Step 2: Attribute-Aware Message-Passing Convolution Attribute-guided message propagation filters irrelevant neighbor signals adaptively according to biological feature consistency. Multi-layer graph convolution aggregates distinctive node representations while suppressing over-smoothing. The orange box corresponds to Step 3: Association score prediction. The aggregated miRNA and disease embeddings are combined to compute matching scores, which represent the likelihood of unvalidated miRNA-disease pairs.

The Attribute-aware Message-Passing Graph Convolutional Network (AAMPGCN), proposed in 2025 [14,53], is an improved graph convolutional model designed to address the over-smoothing problem and inefficient attribute utilization in traditional Graph Convolutional Networks (GCNs). Different from traditional GCNs that only rely on graph structural information for global message passing, AAMPGCN emphasizes the guiding role of node attributes. It assumes that nodes with similar attribute characteristics tend to have similar semantic meanings and interaction patterns, and, thus, high-order message aggregation should be carried out within the scope of these similar nodes. The entire theoretical framework of AAMPGCN consists of three key components: attribute-aware subgraph partitioning, constrained message passing within subgraphs, and multi-subgraph embedding fusion. These components work together to achieve effective integration of node structural information and attribute information, thereby improving the performance of graph learning tasks. This mechanism not only filters out heterogeneous noise caused by cross-attribute propagation but also maintains the discriminability of node embeddings during deep convolution, making it particularly suitable for sparse and heterogeneous networks such as biological information networks. The flowchart of the AAMPGCN model for predicting MDAs is shown in Figure 8.

#### 4.6.1. Attribute-Aware Subgraph Partitioning

To facilitate the derivation of formulas, the following definitions and symbols are specified for the graph structure and node characteristics involved in AAMPGCN:

Let G=(V,E,X) denote the input graph, where $V=\{v_{1},v_{2},\ldots ,v_{N}\}$ is the set of nodes (with N being the total number of nodes), E⊆V×V is the set of edges representing the connections between nodes, and $X\in R^{N\times D}$ is the node attribute matrix (with D being the dimension of node attributes).

Let $S=\{S_{1},S_{2},\ldots ,S_{K}\}$ represent the set of attribute-homogeneous subgraphs obtained by attribute clustering, where K is the number of subgraphs, $S_{k}\subseteq V$ is the set of nodes in the k-th subgraph, and ${⋃}_{k=1}^{K}S_{k}=V$, $S_{i}\cap S_{j}=\emptyset$ for i≠j.

Let $A\in R^{N\times N}$ be the adjacency matrix of the original graph, where $A_{ij}=1$ if $(v_{i},v_{j})\in E$; otherwise, $A_{ij}=0$.

Let $A_{k}\in R^{N\times N}$ be the adjacency matrix of the k-th subgraph, where $A_{k,ij}=1$ if $(v_{i},v_{j})\in E$ and $v_{i},v_{j}\in S_{k}$; otherwise, $A_{k,ij}=0$.

Let $H^{\left(l\right)}\in R^{N\times F_{l}}$ denote the node embedding matrix of the l-th layer of AAMPGCN, where $F_{l}$ is the dimension of node embeddings in the l-th layer. Specifically, $H^{\left(0\right)}=X$ (the initial embedding is the node attribute matrix).

Let $W^{\left(l\right)}\in R^{F_{l-1}\times F_{l}}$ be the trainable weight matrix of the l-th layer, and σ(⋅) be the activation function (e.g., ReLU, Sigmoid).

The core of attribute-aware subgraph partitioning is to group nodes with similar attribute characteristics into the same subgraph. AAMPGCN first maps the original node attributes to a low-dimensional feature space through an attribute encoder to enhance the discriminability of attribute features. The attribute encoder is defined as a two-layer fully connected network:

(14) $$Z=\sigma_{2}(W_{2}\cdot \sigma_{1}(W_{1}\cdot X+b_{1})+b_{2})$$

where $Z\in R^{N\times F_{a}}$ is the low-dimensional attribute embedding matrix (with $F_{a}$ being the dimension of attribute embeddings), $W_{1}\in R^{D\times F_{a}}$ and $W_{2}\in R^{F_{a}\times F_{a}}$ are the weight matrices of the attribute encoder, $b_{1}$ and $b_{2}$ are the bias terms, and $\sigma_{1},\sigma_{2}$ are activation functions (usually ReLU).

On the basis of attribute embeddings Z, K-means clustering is used to divide nodes into K subgraphs. The clustering objective is to minimize the within-cluster sum of squares (WCSS):

(15) $$\underset{S_{1},\ldots ,S_{K}}{min}\sum_{k=1}^{K}\sum_{v_{i}\in S_{k}}∥z_{i}-\mu_{k}{∥}_{2}^{2}$$

where $z_{i}$ is the attribute embedding of the node $v_{i}$, and $\mu_{k}=\frac{1}{\left|S_{k}\right|}\sum_{v_{i}\in S_{k}}z_{i}$ is the centroid of the k-th subgraph. After clustering, we obtain the subgraph partition result S and the corresponding subgraph adjacency matrices $\left\{A_{1},A_{2},\ldots ,A_{K}\right\}$.

#### 4.6.2. Constrained Message Passing Within Subgraphs

Unlike traditional GCNs that perform global message passing on the entire graph, AAMPGCN restricts message propagation within each attribute-homogeneous subgraph. This ensures that the message aggregation process only occurs between nodes with similar attributes, thereby reducing cross-attribute noise and alleviating over-smoothing. The message passing formula for the l-th layer in the k-th subgraph is

(16) Hk(l)=σ(A^k⋅H(l−1)⋅W(l))

where $H_{k}^{\left(l\right)}\in R^{N\times F_{l}}$ is the node embedding matrix of the l-th layer in the k-th subgraph, and A^k is the normalized adjacency matrix of the k-th subgraph. The normalization of the adjacency matrix follows the symmetric normalization method used in traditional GCNs, which avoids the problem of numerical instability caused by excessive node degrees:

(17) A^k=Dk−1/2⋅(Ak+I)⋅Dk−1/2

where $I\in R^{N\times N}$ is the identity matrix (used to retain the self-information of nodes), and $D_{k}\in R^{N\times N}$ is the degree matrix of the k-th subgraph, with $D_{k,ii}=\sum_{j=1}^{N}A_{k,ij}+1$ (the “+1” is due to the addition of the identity matrix).

For nodes not belonging to the k-th subgraph ($v_{i}\notin S_{k}$), their embeddings in $H_{k}^{\left(l\right)}$ are set to 0, indicating that no message propagation occurs for these nodes in the k-th subgraph.

#### 4.6.3. Multi-Subgraph Embedding Fusion

After completing the high-order message passing within each subgraph, AAMPGCN fuses the node embeddings obtained from all subgraphs to generate the final node embeddings. To fully utilize the information of each subgraph, a weighted fusion strategy is adopted, where the weight of each subgraph is determined by the similarity between the node’s attribute embedding and the subgraph’s centroid. The fusion formula is

(18) $$H^{\left(l\right)}=\sum_{k=1}^{K}\alpha_{k,i}\cdot H_{k,i}^{\left(l\right)}$$

where $H_{k,i}^{\left(l\right)}$ is the embedding of the node $v_{i}$ in the k-th subgraph of the l-th layer, and $\alpha_{k,i}$ is the fusion weight of the k-th subgraph for the node $v_{i}$. The weight $\alpha_{k,i}$ is calculated using the softmax function to ensure that the sum of weights for each node is 1:

(19) $$\alpha_{k,i}=\frac{\exp\left(-∥z_{i}-\mu_{k}{∥}_{2}^{2}\right)}{\sum_{m=1}^{K}exp(-∥z_{i}-\mu_{m}{∥}_{2}^{2})}$$

The weight calculation reflects that nodes with higher similarity to the centroid of a subgraph will have a higher weight for that subgraph’s embedding, which ensures that the fused embedding can fully retain the attribute characteristics of the node.

#### 4.6.4. Final Output and Loss Function

After L layers of subgraph message passing and embedding fusion, the final node embedding matrix $H^{\left(L\right)}$ is obtained. For node classification or link prediction tasks (such as miRNA–disease association prediction), a predictor (usually a fully connected network) is used to map the final embeddings to the task output space:

(20) Y^=softmax(Wpred⋅H(L)+bpred)

where Y^∈RN×C is the predicted output matrix (with C being the number of categories or the dimension of the link prediction output), $W_{\mathrm{pred}}$ and $b_{\mathrm{pred}}$ are the weight and bias of the predictor, respectively.

The loss function of AAMPGCN is designed according to the specific task. For classification tasks (such as node type classification), the cross-entropy loss is used:

(21) L=−1N∑i=1N∑c=1CYi,c⋅log(Y^i,c)

where $Y\in R^{N\times C}$ is the true label matrix. For link prediction tasks (such as miRNA–disease association prediction), the binary cross-entropy loss is used:

(22) L=−1M∑(i,j)∈E∪E−(Yij⋅log(Y^ij)+(1−Yij)⋅log(1−Y^ij))

where M is the total number of positive and negative samples, $E^{-}$ is the set of negative edges, $Y_{ij}=1$ if $(v_{i},v_{j})\in E$ (positive sample), and $Y_{ij}=0$ if $(v_{i},v_{j})\in E^{-}$ (negative sample).

#### 4.6.5. Performance Evaluation

In order to evaluate the effectiveness of the proposed computational model, the following indices were adopted as the predictive performance, including accuracy (*Acc*), Recall (*Rec*) and Precision (*Pre*) and *F*1-measure (*F*1), which was defined as follows:

(23) $$Acc=\frac{TP+TN}{TP+FP+TN+FN}$$

(24) $$Rec=\frac{TP}{TP+FN}$$

(25) $$Pre=\frac{TP}{FP+TP}$$

(26) $$F1=\frac{2\times Pre\times Rec}{Pre+Rec}$$

The confusion matrix consists of four basic terms: True Positive (*TP*), True Negative (*TN*), False Positive (*FP*), and False Negative (*FN*). TP denotes samples that are actually positive and predicted as positive; *TN* represents samples that are actually negative and predicted as negative; *FP* refers to samples that are actually negative but misclassified as positive; *FN* indicates samples that are actually positive but misclassified as negative. Based on the four metrics, Precision measures the proportion of correctly predicted positive samples among all predicted positive samples. Recall (Sensitivity) reflects the proportion of correctly identified positive samples among all real positive samples. *F*1-score is the harmonic mean of Precision and Recall, comprehensively evaluating the overall classification performance.

## 5. Conclusions

Accurate prediction of miRNA–disease associations (MDAs) is crucial for deciphering the molecular mechanisms of complex diseases and identifying potential diagnostic biomarkers and therapeutic targets. This study proposed the AAMPGCN model for predicting miRNA–disease associations, aiming to improve the accuracy and reliability of MDA prediction. The superior performance of AAMPGCN is attributed to its core design features: integrating miRNA and disease attribute information into the message-passing process, partitioning attribute-homogeneous subgraphs, and restricting high-order message propagation within subgraphs, which effectively filters cross-attribute noise, avoids over-smoothing, and maintains the discriminability of node embeddings. Moreover, the AAMPGCN prioritizes miRNA and disease attributes, aggregating messages specifically among nodes with similar attribute characteristics that are relevant to miRNA–disease interactions. Case studies further confirmed that the predicted MDAs are biologically meaningful, with all top predicted associations supported by authoritative databases or published experimental literature, verifying the model’s practical application value. In conclusion, AAMPGCN provides a reliable, efficient computational tool for MDA prediction, offering important support for in-depth research on disease pathogenesis, biomarker discovery, and clinical precision medicine. It not only enriches the methodological system of MDA prediction but also lays a solid foundation for subsequent experimental verification and clinical translation of miRNA-related research.

## Figures and Tables

**Table 1:** AAMPGCN evaluation on HMDD v2.0 via five-fold cross-validation.

Testing Set	Acc. (%)	Rec. (%)	Pre. (%)	F1 (%)
1	86.97	88.21	86.67	87.13
2	87.11	89.13	85.66	87.36
3	86.62	88.12	85.82	86.65
4	87.48	89.78	86.83	87.76
5	86.93	86.92	86.76	86.84
Mean	87.02 ± 0.31	88.43 ± 1.09	86.35 ± 0.56	87.15 ± 0.43

**Table 2:** Comparative evaluation of model performance.

Model	AUC (%)	Acc. (%)	Rec. (%)	Pre. (%)	F1 (%)
HHOMR	93.28	86.25	— —	85.54	85.68
MTLMDA	93.05	85.62	84.60	84.81	84.58
VGAEMDA	93.85	87.13	91.03	84.85	87.60
GBDT-LR	92.74	83.04	89.31	83.15	83.02
AAMPGCN	94.06	87.02	88.43	86.35	87.15

**Table 3:** The total number of database-validated miRNA–disease associations among the top 50 predicted entries of each prediction model.

Model	Lung Tumors	Prostate Tumors
HOMR	37	43
GCCDN [44]	-	47
GBDT-LR	-	44
AAMPGCN	46	49

## Data Availability

The datasets that support the findings of this study are available at https://xzcpcumt.cn:8888/downdata.html (accessed on 10 June 2026).

## Abbreviations

GCN	Graph Convolutional Network
AAMPGCN	Associations use Attribute-Aware Message Passing GCN
AUC	Area Under the Curve
AUPR	Area Under the Precision–Recall Curve
MDAs	MiRNA–Disease Associations
ROC	Receiver Operating Characteristic
PR	Precision–Recall
FS	Functional Similarity
MISIM	miRNA Similarity
DAG	Directed Acyclic Graph
GIP	Gaussian Interaction Profile
